# Cardiac Gene Activation Analysis in Mammalian Non-Myoblasic Cells by Nkx2-5, Tbx5, Gata4 and Myocd

**DOI:** 10.1371/journal.pone.0048028

**Published:** 2012-10-29

**Authors:** Lei Zhou, Yu Liu, Li Lu, Xinzheng Lu, Richard A. F. Dixon

**Affiliations:** 1 Department of Molecular Cardiology, Texas Heart Institute, Houston, Texas, United States of America; 2 Department of Biology and Biochemistry, University of Houston, Houston, Texas, United States of America; 3 Department of Biochemistry and Molecular Biology, University of Texas, M. D. Anderson Cancer Center, Houston, Texas, United States of America; 4 Department of Cardiology, the First Affiliated Hospital of Nanjing Medical University, Nanjing, Jiangsu Province, China; Univeristy of California Riverside, United States of America

## Abstract

Cardiac transcription factors are master regulators during heart development. Some were shown to transdifferentiate tail tip and cardiac fibroblasts into cardiomyocytes. However, recent studies have showed that controversies exist. Potential difference in tail tip and cardiac fibroblast isolation may possibly confound the observations. Moreover, due to the use of a cardiac reporter (Myh6) selection strategy for induced cardiomyocyte enrichment, and the lack of tracking signals for each transcription factors, individual roles of each transcription factors in activating cardiac gene expression in mammalian non-myoblastic cells have never been elucidated. Answers to these questions are an important step toward cardiomyocyte regeneration. Because mouse 10T1/2 fibroblasts are non-myoblastic in nature and can be induced to express genes of all three types of muscle cells, they are an ideal model for the analysis of cardiac and non-cardiac gene activation after induction. We constructed bi-cistronic lentiviral vectors, capable of expressing cardiac transcription factors along with different fluorescent tracking signals. By infecting 10T1/2 fibroblasts with Nkx2-5, Tbx5, Gata4 or Myocd cardiac transcription factor lentivirus alone or different combinations, we found that only Tbx5+Myocd and Tbx5+Gata4+Myocd combinations induced Myh6 and Tnnt2 cardiac marker protein expression. Microarray-based gene ontology analysis revealed that Tbx5 alone activated genes involved in the Wnt receptor signaling pathway and inhibited genes involved in a number of cardiac-related processes. Myocd alone activated genes involved in a number of cardiac-related processes and inhibited genes involved in the Wnt receptor signaling pathway and non-cardiac processes. Gata4 alone inhibited genes involved in non-cardiac processes. Tbx5+Gata4+Myocd was the most effective activator of genes associated with cardiac-related processes. Unlike Tbx5, Gata4, Myocd alone or Tbx5+Myocd, Tbx5+Gata4+Myocd activated the fewest genes associated with non-cardiac processes. Conclusively, Tbx5, Gata4 and Myocd play different roles in cardiac gene activation in mammalian non-myoblastic cells. Tbx5+Gata4+Myocd activates the most cardiac and the least non-cardiac gene expression.

## Introduction

Cardiac transcription factors Nkx2.5, Tbx5, Gata4, and Myocd are master regulators during heart development. The deletion of any of these genes results in the failure of normal heart development and cardiomyocyte maintenance [1]–[5]. Recently, the combination of Tbx5+Gata4+Smarcd3 was shown to transdifferentiate non-cardiac mesoderm cells into cardiomyocytes [6], and the combinations of Tbx5+Gata4+Mef2c and Tbx5+Gata4+Mef2c+Hand2 were shown to transdifferentiate tail tip and cardiac fibroblasts into cardiomyocytes [7]–[9], making customized cardiomyocyte regeneration possible. However, a more recent study reported that Tbx5+Gata4+Mef2c is inefficient for reprogramming tail tip and cardiac fibroblasts into cardiomyocytes, as determined by molecular and electrophysiological characterization [10]. Furthermore, in the two independent studies [7], [9], the cardiac reprogramming abilities of Tbx5+Gata4+Mef2c and Tbx5+Gata4+Mef2c+Hand2 combinations have been compared side by side. However, the conclusions have differed, adding complexity and confusion to the current understanding of cardiac transcription factor-based cardiac reprogramming. Potential difference in tail tip and cardiac fibroblast isolation may be a reason for these confounding observations. Moreover, due to the use of a cardiac reporter (Myh6) selection strategy for induced cardiomyocyte enrichment and the lack of tracking signals for each cardiac transcription factors in previous studies, the individual roles of each cardiac transcription factors in activating cardiac gene expression in mammalian non-myoblastic cells have never been elucidated. Answers to these questions are an important step toward cardiomyocyte regeneration.

We therefore choose the mouse 10T1/2 fibroblast cell line to evaluate the ability of several important cardiac transcription factors to activate cardiac genes. 10T1/2 cells are derived from mouse embryonic cells with fibroblast morphology. Because 10T1/2 cells are non-myoblastic in nature [11] but possess the potential to be induced to express genes of all three types of muscle cells (skeletal, smooth and cardiac) [12]–[16], these cells are an ideal model for the analysis of cardiac and non-cardiac gene activation after treatment with cardiac transcription factor. We constructed internal ribosome entry site (IRES)-mediated bi-cistronic lentiviral vectors, capable of expressing cardiac transcription factors along with sub-cellular localized fluorescent tracking signals in the transfected cells, so that individual roles of each cardiac transcription factors can be determined. Among the tested cardiac transcription factors, Nkx2-5 is the earliest known marker of vertebrate heart development [1]. Tbx5 and Gata4 are common constituents of the transcription factor combinations recently reported to induce cardiac transdifferentiation: Tbx5+Gata4+Smarcd3 [6], Tbx5+Gata4+Mef2c [7], [8], and Tbx5+Gata4+Mef2c+Hand2 [9]. Myocd is a highly potent cardiac transcription co-activator that functions through serum response factor (Srf) [4]. Previously, physical interactions with functional significance have been shown between Tbx5 and Gata4 [17], Tbx5 and Myocd [18], Nkx2-5 and Gata4 [19], and Nkx2-5 and Tbx5 [20].

After infecting 10T1/2 fibroblasts with human Nkx2-5 (N), Tbx5 (T), Gata4 (G), or Myocd (M) lentivirus, either alone or in different combinations, we examined cardiac and non-cardiac (e.g. skeletal muscle, smooth muscle) gene activation in these cells. Here we show that only the combinations of T+M and T+G+M can induce Myh6 and Tnnt2 cardiac marker gene expression *de novo*. Tbx5, Gata4, and Myocd contributed differently to cardiac gene activation and non-cardiac gene suppression. The combination of Tbx5+Gata4+Myocd was the most effective activator of genes associated with cardiac-related processes including muscle cell differentiation, sarcomere, striated muscle contraction, and regulation of heart contraction.

## Materials and Methods

### Construction of Nkx2-5, Tbx5, Gata4 and Myocd lentiviruses expressing different fluorescent tracking signals

Tet-on lentiviral vector (pNLTREpitt-EGFP-ΔU3) and Tet transactivator vector (pNLrtTA2sM2) were provided by Dr. Jakob Reiser [21]. An XhoI/NsiI blunted fragment from pMSCVpuro (Clontech) was inserted into the blunted BamHI site of pNLrtTA2sM2 to generate pNLrtTA2sM2-puro for selection of a Tet transactivator cell line. pNLTREpitt-EGFP-ΔU3 was used as a backbone to generate Tet-on bicistronic lentiviruses that express cardiac transcription factors as well as different fluorescent tracking signals. Briefly, complementary deoxyribonucleic acids (cDNAs) of human Nkx2-5 (OriGene) and peroxisome Discosoma sp. red fluorescent protein variant MST (DsRed-MST), human Tbx5 (OriGene) and nuclear DsRed-MST, human Gata4 (OriGene) and peroxisome enhanced green fluorescent protein (EGFP), and human Myocd (accession number AY764180, provided by Dr. Antoine A.F. de Vries) [22] and nuclear EGFP were linked together with an encephalomyocarditis virus-derived IRES sequence, respectively. These fragments were inserted into the blunted BamHI/BsrGI site of pNLTREpitt-EGFP-ΔU3. The resulting constructs were TRE-Nkx2-5-IPR, TRE-Tbx5-INR, TRE-Gata4-IPG, and TRE-Myocd-ING. IPR, INR, IPG, and ING indicate IRES peroxisomal DsRed-MST, IRES nuclear DsRed-MST, IRES peroxisomal EGFP, and IRES nuclear EGFP, respectively. LacZ controls (TRE-LacZ-IPR, TRE-LacZ-INR, TRE-LacZ-IPG, and TRE-LacZ-ING) were generated accordingly. All lentiviruses were packaged in 293FT cells (Invitrogen) by using pME VSV-G and pHIV-PV plasmids provided by Dr. Richard Sutton [23]. Virus titer was determined by using Lenti-X p24 Rapid Titer Kit (Clontech).

### Luciferase reporters and dual-luciferase assay

The Tbx5 luciferase *cis*-element reporter (2xTBE-Luc) and its mutant (2xmuTBE-Luc) were provided by Dr. Eric Olson [24]. Based on published in vitro site-selection studies [25]–[27], we inserted one or two copies of wild-type (bold) or site-mutated (underlined) binding sequences of Nkx2-5, Gata4, or Srf into pLuc-MCS (Stratagene) at the HindIII/XhoI site.

2xNKE: 5′-CT**GCAAGTG**A**CAGAATGG**GCT**GCAAGTG**A**CAGAATGG**G-3′,

2xmuNKE: 5′-CT**GCATATG**A**CAGCCAGG**GCT**GCATATG**A**CAGCCAGG**G-3′,

GATA site: 5′-CAAAGGGCC**GATG**GGCA**GATA**GAGGAGAGACAGGA-3′,

muGATA site: 5′-CAAAGGGCC**TCAG**GGCA**TGCA**GAGGAGAGACAGGA-3′,

2xSRE: 5′-ACAC**CCAAATATGG**CTACAC**CCAAATATGG**CT-3′,

2xmuSRE: 5′-ACAC**CCA-GATCTG**CTACAC**CCA-GATCTG**CT-3′.

The *cis*-element reporters were named 2xNKE-Luc, 2xmuNKE-Luc, GATA-Luc, muGATA-Luc, 2xSRE-Luc, and 2xmuSRE-Luc, respectively. Nkx2-5, Tbx5, Gata4, and Myocd lentiviruses were characterized in CV-1 fibroblasts (American Type Culture Collection, the most commonly used cell line for the luciferase assay,) by using a dual-luciferase assay system (Promega). For the Myocd lentivirus, Srf expression vector pCGN-SRF or its control vector pCGN (provided by Dr. Robert Schwartz [28]) was added. Up to four kinds of LacZ control plasmids were added to equalize the total plasmid amount. Doxycycline (1 ug/ml) was added to induce transgene expression. Firefly luciferase activity was normalized to that of the internal control, Renilla luciferase.

### 10T1/2 cell culture and virus infection

The 10T1/2 Tet-transactivator cell line (10T1/2-tTA) was established by infecting 10T1/2 cells (American Type Culture Collection) with pNLrtTA2sM2-puro lentivirus, followed by puromycin selection. 10T1/2-tTA cells were then infected with different combinations of the tetracycline-dependent Nkx2-5, Tbx5, Gata4, and Myocd lentiviruses. To induce transgene expression, doxycycline was added at a final concentration of 1 µg/ml.

### Immunofluorescence and confocal microscopy

10T1/2-tTA cells that were infected with different combinations of Nkx2-5, Tbx5, Gata4, and Myocd lentiviruses were seeded on glass cover slips and grown in Basal Medium Eagle (BME) medium (Invitrogen) supplemented with 1 µg/ml doxycycline, 5% (v/v) horse serum (Invitrogen), and 2 mM L-glutamine. Ten days after doxycycline induction, cells were stained with monoclonal antibodies against Myh6 (MF20, The Developmental Studies Hybridoma Bank) or Tnnt2 (Abcam) and Alexa Fluor 350-conjugated goat anti-mouse IgG (Invitrogen). Images were collected by using an Olympus FluoView FV1000 confocal laser scanning microscope. DAPI (4′,6-diamidino-2-phenylindole) nuclear staining was used when needed. Gene cotransfection efficiency was determined as the multitransgene-positive cell number divided by the total cell number. The induction rate of Myh6− or Tnnt2-expressing cells was determined as the percentage of Myh6^+^ or Tnnt2^+^ cells within a designated multitransgene-positive cell population.

### Microarray and gene ontology analysis

10T1/2-tTA cells that were infected with different combinations of Tbx5, Gata4, and Myocd lentiviruses were grown in BME medium supplemented with 1 µg/ml doxycycline, 5% (v/v) horse serum (Invitrogen), and 2 mM L-glutamine. Forty-eight hours later, positively transfected cells were sorted out by using FACS Vantage SE DiVa system (BD Biosciences). Because peroxisomal EGFP (tracking for Gata4) and nuclear EGFP (tracking for Myocd) are of the same excitation/emission wavelength but with different subcellular localization ability, Gata4^+^Myocd^+^ and Tbx5^+^Gata4^+^Myocd^+^ cell purities were determined under a confocal microscope after cell sorting. Sorted cells were then plated in 60 mm dishes and grown in BME medium supplemented with 1 µg/ml doxycycline, 5% (v/v) horse serum (Invitrogen), and 2 mM L-glutamine for an additional 12 days. Samples were from three independent experiments, and total RNA was isolated by using Trizol reagent (Invitrogen), followed by DNase I (Qiagen) treatment. Samples were compared by using Affymetrix MG 430 2.0 arrays. All data are Minimum Information About a Microarray Experiment (MIAME) compliant and the raw data have been deposited in the Gene Expression Omnibus (GEO) database (accession number GSE27329). Expression data were analyzed by using dChip2008 [29] and GenMAPP [30] software programs. Activated or inhibited genes were defined as genes with an absolute fold change ≥2.0 relative to the LacZ group and genes with a signal intensity equals to 0 in the LacZ group but not in the transcription factor-treated groups or vice versa. A *p* value cut-off of 0.05 was used. Activated or inhibited gene lists were compared by using the GeneVenn web application [31]. Permutation test was applied for multiple testing correction, and a permute *p* value was calculated by using a non-parametric bootstrapping approach [30]. GO terms with a permute *p*<0.05 and a nested gene change number ≥4 were considered as significantly activated or inhibited.

### Statistical analysis

All results were expressed as mean ± standard error of the mean. Statistical significance was evaluated by analysis of variance following one-way ANOVA procedure with SPSS11.0 software (SPSS Inc.). A value of *p*<0.05 was considered statistically significant.

## Results

### Nkx2-5, Tbx5, Gata4, and Myocd lentiviral vectors transactivated *cis*-element reporters containing cognate sequences of Nkx2-5, Tbx5, Gata4, and Srf binding sites

**Figure 1:**
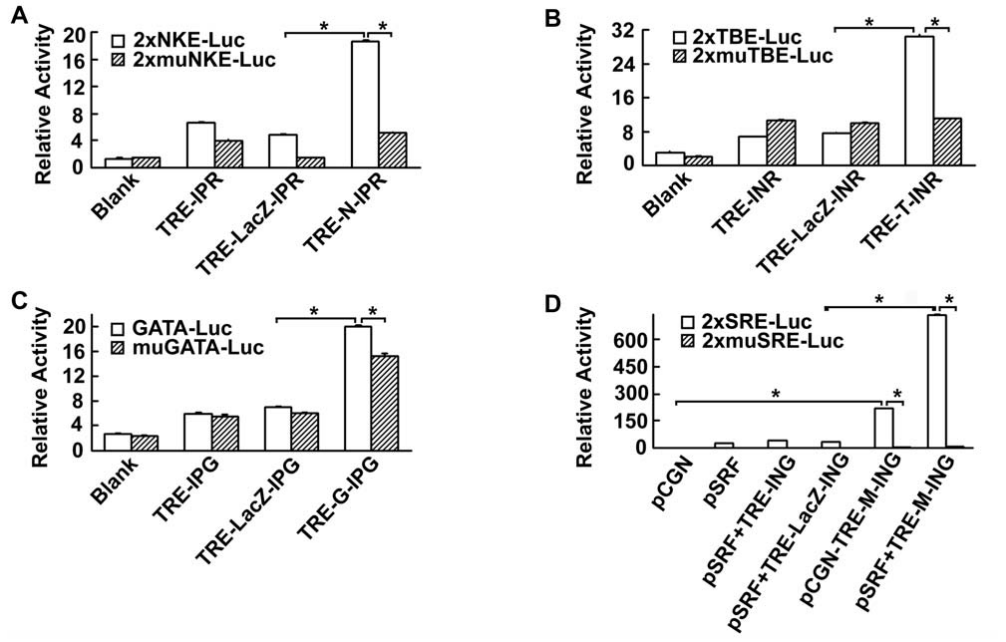
Nkx2-5, Tbx5, Gata4 and Myocd lentiviral vectors transactivated *cis*-element reporters containing cognate sequences of Nkx2-5, Tbx5, Gata4, and Srf binding sites. CV-1 cells were cotransfected with Tet-transactivator vector, corresponding luciferase reporters, and one or more of Nkx2-5, Tbx5, Gata4, Myocd, or LacZ tetracycline-dependent lentiviral vectors. Dual-luciferase assays were performed 48 hours after doxycycline induction. (**A**) TRE-Nkx2-5-IPR transactivated NKE *cis*-element reporter. (**B**) TRE-Tbx5-INR transactivated TBE *cis*-element reporter. (**C**) TRE-Gata4-IPG transactivated GATA *cis*-element reporter. (**D**) TRE-Myocd-ING transactivated SRE *cis*-element reporter in the presence of Srf. Each experiment was performed in quadruplicate. Blank, without the addition of tetracycline-dependent lentiviral vector; TRE-IPR, TRE-INR, TRE-IPG, and TRE-ING are empty vector controls for Nkx2-5, Tbx5, Gata4, and Myocd lentiviruses. TRE-LacZ-IPR, TRE-LacZ-INR, TRE-LacZ-IPG, and TRE-LacZ-ING are LacZ vector controls for Nkx2-5, Tbx5, Gata4, and Myocd lentiviruses. TRE-N-IPR, TRE-T-INR, TRE-G-IPG, and TRE-M-ING are Nkx2-5, Tbx5, Gata4, and Myocd lentiviruses. *P<0.01 vs LacZ groups.

The transactivation ability of Nkx2-5, Tbx5, Gata4, and Myocd lentiviral vectors was characterized by using *cis*-element luciferase reporters containing wild-type and mutated cognate sequences of Nkx2-5, Tbx5, Gata4, and Myocd binding sites in CV-1 fibroblasts. Compared with LacZ lentivirus, Nkx2-5 lentivirus significantly transactivated the 2xNKE wild-type reporter (18.63±0.26 vs 4.89±0.09, *p*<0.01) but not the 2xmuNKE mutated reporter (5.17±0.17, *p*<0.01 vs wild type, **Figure 1A**); Tbx5 lentivirus significantly transactivated the 2xTBE wild-type reporter (30.39±0.6 vs 7.60±0.06, *p*<0.01) but not the 2xmuTBE mutated reporter (11.09±0.14, *p*<0.01 vs wild type, **Figure 1B**); and Gata4 lentivirus significantly transactivated the GATA wild-type reporter (20.05±0.19 vs 7.06±0.06, *p*<0.01) but not the muGATA mutated reporter (15.30±0.42, *p*<0.01 vs wild type, **Figure 1C**). Myocd acts as cofactor of Srf, therefore, compared with LacZ lentivirus, Myocd lentivirus in the presence of Srf significantly transactivated the 2xSRE wild-type reporter (736.74±2.73 vs 32.13±0.61, *p*<0.01) but not the site-mutated 2xmSRE reporter (7.90±0.18, *p*<0.01 vs wild type, **Figure 1D**).

Additionally, because Nkx2-5, Tbx5, Gata4, and Myocd lentiviruses were designed to co-express peroxisomal DsRed-MST, nuclear DsRed-MST, peroxisomal EGFP, and nuclear EGFP tracking signals, respectively; multigene transfection within a single cell was able to be tracked under fluorescent microscope by the presence of different subcellular localized signals (**Supplemental Figure S1**).

### Myh6 and Tnnt2 cardiac marker protein expression was only induced by Tbx5+Myocd and Tbx5+Gata4+Myocd combinations in 10T1/2 non-myoblastic cells

**Figure 2:**
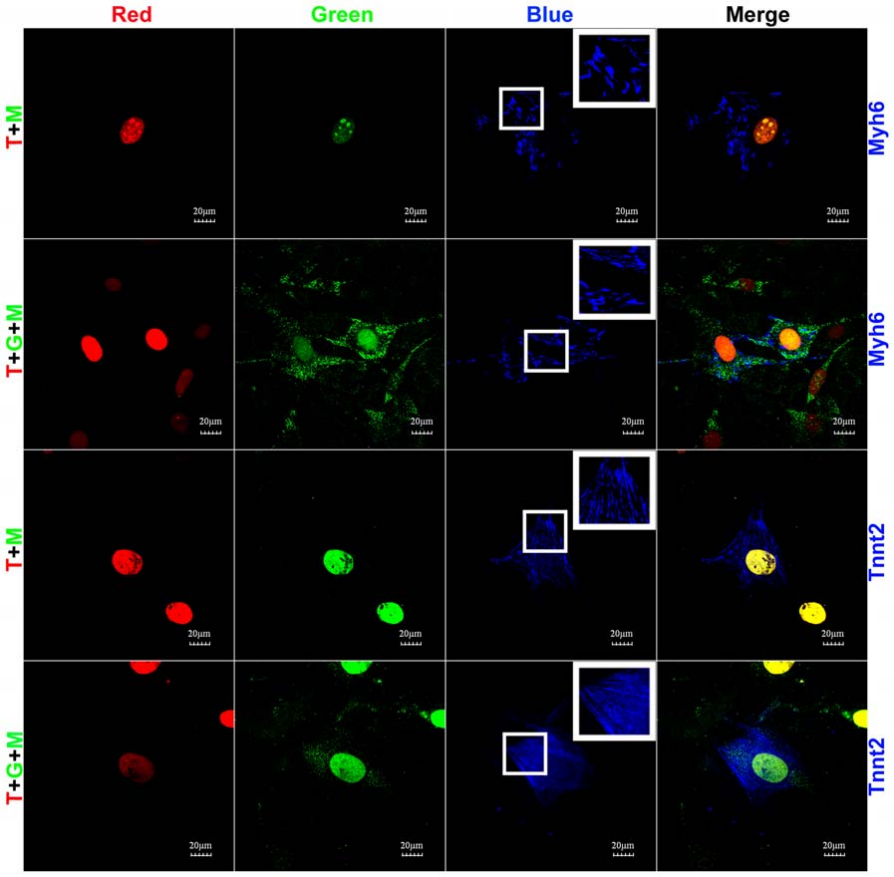
Tbx5+Myocd and Tbx5+Gata4+Myocd combinations induced Myh6 and Tnnt2 cardiac marker protein expression in 10T1/2 non-myoblastic cells. 10T1/2 fibroblasts were infected with Nkx2-5, Tbx5, Gata4, Myocd lentivirus alone, or different combinations. LacZ lentivirus was used as control. Ten days after doxycycline induction, Myh6 and Tnnt2 protein expression was examined by incubating the cells with Myh6 or Tnnt2 primary antibody and was visualized by Alexa Fluor 350-conjugated secondary antibody. Only T+M and T+G+M combinations induced *de novo* Myh6 and Tnnt2 protein expression. Myh6^+^ and Tnnt2^+^ cell induction rates in designated cell populations were summarized in Table 1. Insets showed nascent fibril-like structures. Scale bar = 20 µm. N, Nkx2-5; T, Tbx5; G, Gata4; M, Myocd.

Among 15 different combinations, only the T+M and T+G+M combinations induced *de novo* expression of both Myh6 and Tnnt2 proteins in 10T1/2 non-myoblastic cells. The induced proteins exhibited nascent, fibril-like structures (**Figure 2**). Because not all T^+^M^+^ and T^+^G^+^M^+^ cells expressed Myh6 and Tnnt2 (**Figure 2**), these Myh6^−^/T^+^G^+^M^+^, Tnnt2^−^/T^+^G^+^M^+^, and Tnnt2^−^/T^+^M^+^ cells served as stringent negative controls for Myh6− and Tnnt2-positive immunostaining in T^+^G^+^M^+^ and T^+^M^+^ cells, excluding the possibility of false-positive results. The induction rate of Myh6^+^ cells in Tbx5^+^Myocd^+^ and Tbx5^+^Gata4^+^Myocd^+^ cell populations was 15.7% and 5.9%, respectively (**Table 1**). The induction rate of Tnnt2^+^ cells in Tbx5^+^Myocd^+^ and Tbx5^+^Gata4^+^Myocd^+^ cell populations was 26.1% and 7.4%, respectively (**Table 1**). The molecular weights of the induced Myh6 and Tnnt2 proteins were confirmed to be same as those in mouse heart by Western blot analysis (**Supplemental Figure S2**).

**Table 1:** Multigene transfection efficiency and Myh6^+^ and Tnnt2^+^ cell induction rate.

Gene(s)	Multigene transfection efficiency‡ §	Myh6^+^ cell induction rate‡ ∥	Tnnt2^+^ cell induction rate‡ ∥
**LacZ**	10.01±0.54	0	0
**N**	37.51±1.96	0	0
**T**	10.11±1.21	0	0
**G**	92.75±2.38	0	0
**M**	7.18±1.16	0	0
**N+T**	8.25±0.32	0	0
**N+G**	37.34±2.02	0	0
**N+M**	3.04±1.47	0	0
**T+G**	9.24±1.71	0	0
**T+M**	1.69±0.07	15.70±0.66	26.10±0.72
**G+M**	11.95±0.97	0	0
**N+T+G**	11.73±0.48	0	0
**N+T+M**	1.56±0.05	0	0
**N+G+M**	0.06±0.01*	0†	0†
**T+G+M**	3.78±0.36	5.87±0.47	7.40±0.62
**N+T+G+M**	0.05±0.01*	0†	0†

* >30,000 total cells per group were analyzed.

† >100 multigene-transfected cells were analyzed.

‡ Data are expressed as (mean ± standard error of mean)%.

§ More than 1000 total cells per group were analyzed to determine multigene transfection efficiency.

∥ More than 200 multigene-transfected cells were analyzed to determine Myh6 and Tnnt2 induction rate.

N, Nkx2-5; T, Tbx5; G, Gata4; M, Myocd.

### Tbx5+Gata4+Myocd activated cardiac genes more specifically than did Tbx5+Myocd

**Figure 3:**
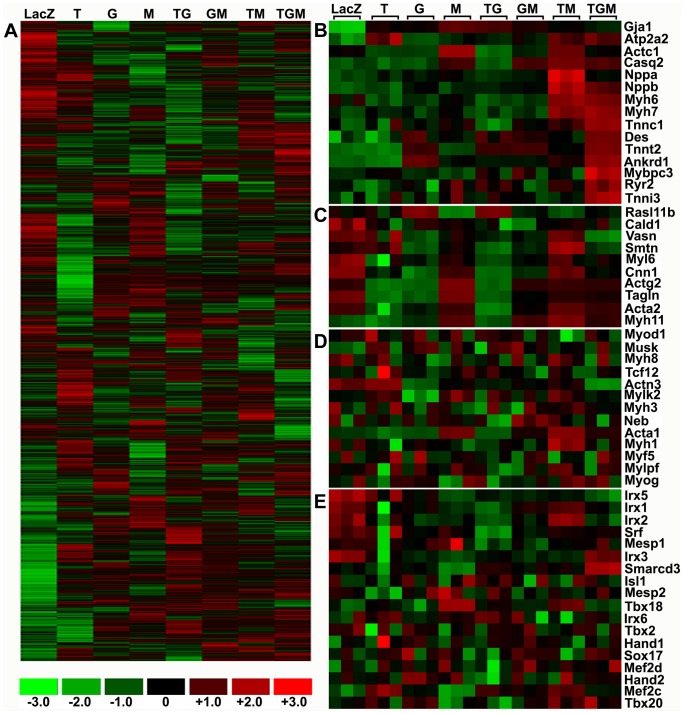
Tbx5+Gata4+Myocd was the most effective activator of cardiac genes. 10T1/2 fibroblasts were infected with Tbx5, Gata4, Myocd lentivirus alone, or different combinations. LacZ lentivirus was used as control. Genetically unbiased genome-wide expression profiles were obtained 14 days after doxycycline induction. (**A**) Global gene expression. (**B**) Cardiac structural gene enrichment. (**C**) Smooth muscle gene enrichment. (**D**) Skeletal muscle gene enrichment. (**E**) Cardiac-related transcription factor enrichment. Signal values were log_2_ transformed. Signal values below 0 were set to 1. Each group included biological triplicates. T, Tbx5; G, Gata4; M, Myocd; TG, T+G; GM, G+M; TM, T+M; TGM, T+G+M.

To determine the difference between T+M- and T+G+M-activated cardiac gene expression and to determine the individual roles of Tbx5, Gata4, and Myocd in activating cardiac gene expression in mammalian non-myoblastic cells, we conducted genome-wide gene expression analysis by using 10T1/2 fibroblasts transfected with Tbx5, Gata4, Myocd alone, or different combinations of these factors. The purity of Gata4^+^Myocd^+^ and Tbx5^+^Gata4^+^Myocd^+^ cells used was 78.1% and 57.4%, respectively. Generally, Tbx5, Gata4, Myocd alone, and different combinations of these factors extensively perturbed whole-genome gene expression (**Figure 3A**). The number of activated genes was 907(T), 812(G), 712(M), 1095(T+G), 884(G+M), 908(T+M), and 878(T+G+M) (**Supplemental Table S1**). The number of inhibited genes was 833(T), 767(G), 616(M), 1041(T+G), 508(G+M), 524(T+M), and 713(T+G+M) (**Supplemental Table S1**). Enriched cardiac, smooth muscle and skeletal muscle gene expression profiles are shown in **Figure 3B–D**. T+G+M induced expression of all cardiac structural genes, including Myh6, Myh7, Actc1, Tnni3, Tnnt2, Tnnc1, Atp2a2, Ryr2, Casq2, Gja1, Des, Mybpc3, Ankrd1, Nppa, and Nppb. T+M induced expression of all cardiac structural genes except Mybpc3 and Tnni3 (**Figure 3B**). Array signal values of several known cardiac marker genes (Myh6, Tnnt2, Ryr2, and Nppa) are shown in **Supplemental Table S2**, and their expression was further validated by quantitative reverse-transcription polymerase chain reaction (qRT-PCR) analysis (**Supplemental Figure S3**). T+M induced Myh11 and Smtn smooth muscle gene expression (29.08- and 1.95-fold increase, respectively), whereas T+G+M only induced relatively lower Myh11 expression (8.42-fold increase, **Figure 3C**). T+M also induced Acta1 and Myh1 skeletal muscle gene expression (80.22- and 1.70-fold increase) whereas T+G+M induced much lower Acta1 expression (9.0-fold increase, **Figure 3D**). The cardiogenic transcription factor expression profile (**Figure 3E**) showed that T+G+M moderately upregulated the expression of Tbx20 (1.99-fold increase) and Smarcd3 (1.69-fold increase). T+M activated Mef2c expression (2.73-fold increase) and moderately upregulated Tbx20 (1.80-fold increase) and Tbx18 (1.44-fold increase) expression. Little change was seen in Hand2 expression after T+M and T+G+M induction. Cardiac mesoderm marker Mesp1 and second-heart field marker Isl1 were not activated by T+M or T+G+M. Nkx2-5, Tbx5, Gata4, and Myocd are normally expressed in adult mouse heart, with Nkx2-5 being expressed the least (**Supplemental Figure S4**). However, endogenous Nkx2-5, Tbx5, Gata4, and Myocd were neither activated nor inhibited by T+M or T+G+M (array signal values are shown in **Supplemental Table S3**).

Gene ontology analysis was summarized in **Table 2**, **Table 3**, and **Table 4**. Perturbation of common biologic processes (e.g. DNA integrity checkpoint, DNA replication initiation, ubiquitin cycle, cell ion homeostasis, protein biosynthesis, regulation of transcription) was not included. The results showed that Tbx5 alone activated genes involved in the Wnt receptor signaling pathway and in several non-cardiac processes (e.g. visual perception, gastrulation, neuron development, and complement activation; **Table 2**). Tbx5 alone inhibited genes involved in several cardiac-related processes (e.g. sarcomere, myofibril, muscle development, muscle contraction, circulation; **Table 3**). Myocd alone activated genes involved in several cardiac-related processes (e.g. circulation, contractile fiber, muscle contraction, sarcomere, myofibril, muscle cell differentiation) and non-cardiac processes (e.g. complement activation, morphogenesis of an epithelium, bone mineralization, morphogenesis of a branching structure, axon guidance; **Table 2**). Myocd alone inhibited genes involved in the Wnt receptor signaling pathway and several non-cardiac processes (e.g. reproduction, epidermis development, ectoderm development, neuron development; **Table 3** **and** **Table 4**). Gata4 alone activated genes involved in circulation and fatty acid oxidation processes and several non-cardiac processes (complement activation, morphogenesis of an epithelium, epithelial cell differentiation, regulation of myeloid cell differentiation; **Table 2**). Gata4 alone inhibited genes involved in several cardiac-related processes (e.g. sarcomere, myofibril, contractile fiber and muscle contraction; **Table 3**) and non-cardiac processes (e.g. tube morphogenesis, epidermis development, ectoderm development, neuron development, meiotic cell cycle; **Table 4**).

**Table 2:** Cardiac and non-cardiac gene processes activated by Tbx5, Gata4, and Myocd.

Gene ontology name	T	G	M	T+G	G+M	T+M	T+G+M
Cardiac	*(permute P value)*						
Wnt receptor signaling pathway	0.004*	N	N	0.63	1.0	0.602	0.802
Circulation	0.54	0.016*	0*	0.01*	0.015*	0*	0*
Contractile fiber	N	N	0.01*	N	N	0.001*	0.002*
Muscle contraction	N	N	0.002*	0.767	0.126	0*	0.006*
Muscle development	0.213	0.803	0.016*	1.0	0.469	0.077	0.032*
Myofibril	N	N	0.005*	N	N	0*	0.006*
Sarcomere	N	N	0.002*	N	N	0*	0.003*
Structural constituent of cytoskeleton	N	N	0.043*	N	0.518	0.131	0.013*
Muscle cell differentiation	N	N	0.019*	0.089	0.036*	0.007*	0.041*
Striated muscle contraction	N	N	N	N	N	0*	0*
Regulation of heart contraction	N	N	N	N	N	N	0*
Structural constituent of muscle	N	N	N	N	N	N	0.003*
Glucose metabolism	N	0.115	0.586	0.083	0.428	0.417	0.009*
Fatty acid oxidation	N	0.001*	N	0*	0.02*	0.039*	0.046*

Non-cardiac	*(permute P value)*						
Complement activation	0.005*	0.004*	0.001*	0.007*	0.003*	0.002*	0.005*
Regulation of ossification	N	N	0*	N	0.001*	N	0.006*
Morphogenesis of an epithelium	0.27	0.006*	0.048*	0.119	0.267	0.025*	N
Humoral immune response	0.029*	0.001*	0*	0.105	0.008*	0.028*	0.418
Gonad development	N	N	N	0.103	0.04*	N	N
Sensory perception of sound	N	N	N	N	0.049*	0.078	N
Bone mineralization	0.002*	N	0.001*	N	0*	N	N
Morphogenesis of a branching structure	0.071	0.062	0.043*	0.03*	0.013*	0.08	N
Sex differentiation	N	N	N	0.31	0.046*	0.114	N
Axon guidance	0*	0.145	0.009*	0.527	0.016*	0*	0.07
Visual perception	0.034*	0.323	0.077	0.769	0.029*	0.543	0.124
Ossification	0.008*	0.003*	0.002*	0.036*	0*	0.077	0.088
Embryonic pattern specification	N	N	N	0.037*	N	N	N
Blood vessel development	0.532	0.009*	0.068	0.047*	0.127	0.021*	0.131
Epithelial cell differentiation	N	0.005*	N	N	N	N	N
Regulation of myeloid cell differentiation	N	0.037*	N	N	N	N	N
Gastrulation	0.017*	N	N	N	N	N	N
Neuron development	0*	0.826	0.47	1.0	0.183	0.03*	0.518

* GO terms with permute *p*<0.05 and a nested gene change number ≥4 were considered to be significantly activated. GOs with a nested gene change number <4 were shown as “N”. GO terms that were both activated and inhibited were excluded. T, Tbx5; G, Gata4; M, Myocd.

**Table 3:** Cardiac gene processes inhibited by Tbx5, Gata4 and Myocd.

Gene ontology name	T	G	M	T+G	G+M	T+M	T+G+M
Cardiac	*(permute P value)*						
Wnt receptor signaling pathway	1.0	0.196	0.018*	N	N	0.097	0.018*
Heart development	0.796	N	0.014*	N	N	0.029*	N
Myoblast differentiation	N	N	N	0.026*	N	N	N
Sarcomere	0*	0.004*	N	0.006*	N	N	N
Myofibril	0*	0.008*	N	0.015*	N	N	N
Skeletal muscle fiber development	N	N	N	0.023*	N	N	N
Contractile fiber	0*	0.004*	N	0.013*	N	N	N
Structural constituent of cytoskeleton	0.018*	0.07	N	0.019*	N	N	N
Muscle development	0.001*	0.134	0.742	0.009*	0.164	0.514	0.793
Actin cytoskeleton organization and biogenesis	0.006*	0.056	0.617	0.002*	1.0	0.396	0.045*
Cytoskeletal protein binding	0.027*	0.067	0.45	0.013*	0.854	0.69	0.25
Muscle contraction	0.001*	0.003*	0.052	0.004*	0.017*	0.01*	0.189
Circulation	0.018*	0.077	N	0.393	0.104	N	0.483

* GO terms with permute *p*<0.05 and a nested gene change number ≥4 were considered to be significantly inhibited. GOs with a nested gene change number <4 were shown as “N”. GO terms that were both activated and inhibited were excluded. T, Tbx5; G, Gata4; M, Myocd.

**Table 4:** Non-cardiac gene processes inhibited by Tbx5, Gata4 and Myocd.

Gene ontology name	T	G	M	T+G	G+M	T+M	T+G+M
Non-cardiac	*(permute P value)*						
Embryonic limb morphogenesis	N	N	N	N	N	N	0.017*
Embryonic appendage morphogenesis	N	N	N	N	N	N	0.017*
Cartilage development	0*	N	N	0*	0*	0*	0.001*
Blood coagulation	0.001*	0*	0.016*	0.016*	0.024*	N	0.003*
Tube morphogenesis	0.077	0.049*	N	0.06	N	0.006*	0.006*
Epidermis development	0.062	0.002*	0.013*	0*	0*	0.006*	0.008*
Axonogenesis	0.081	0.001*	0*	0.004*	0.003*	0*	0.002*
Ossification	0.777	0.139	0.105	0.161	0.195	0.162	0.029*
Central nervous system development	0.055	N	0.189	0.398	0.152	0.026*	0.013*
Ectoderm development	0.171	0.002*	0.024*	0.001*	0*	0.001*	0.002*
Embryonic development	0.074	0.05	0.018*	0.029*	0.087	0.002*	0.005*
Neuron development	0.417	0.006*	0*	0.034*	0.021*	0*	0.011*
Blood vessel development	0.11	0.47	0.018*	0.41	0.028*	0.004*	0.007*
Skeletal development	0.006*	0*	0*	N	0*	0*	0*
Development	0*	0*	0*	0*	0*	0*	0*
Humoral immune response	N	N	N	0.563	N	N	0.011*
Meiotic cell cycle	0.521	0.007*	N	0.008*	0.007*	N	N
Reproduction	0.085	0.853	0.003*	0.134	0.037*	0.052	0.449
Germ cell development	0.006*	N	0.008*	N	N	N	N
Eye development	0.016*	N	0.009*	0.265	N	N	N
Gonad development	N	N	0.012*	0.065	N	N	N
T cell activation	0.063	0.12	0.033*	0.527	0.062	0.025*	0.094
Female gamete generation	0.002*	N	N	0.058	N	N	N

* GO terms with permute *p*<0.05 and a nested gene change number ≥4 were considered to be significantly inhibited. GOs with a nested gene change number <4 were shown as “N”. GO terms that were both activated and inhibited were excluded. T, Tbx5; G, Gata4; M, Myocd.

The combination of T+G+M activated genes associated with the most cardiac-related processes, including muscle development, muscle cell differentiation, structural constituent of cytoskeleton, structural constituent of muscle, myofibril, sarcomere, contractile fiber, muscle contraction, striated muscle contraction, regulation of heart contraction, circulation, glucose metabolism and fatty acid oxidation (two major forms of cardiomyocyte energy metabolism; **Table 2**). Moreover, T+G+M did not activate genes involved in the Wnt receptor signaling pathway and non-cardiac processes (e.g. morphogenesis of an epithelium, bone mineralization, morphogenesis of a branching structure, ossification, axon guidance, visual perception, gastrulation), which were activated by Tbx5, Gata4, or Myocd alone (**Table 2**). T+G+M inhibited genes associated with many non-cardiac processes (e.g. embryonic limb development, blood coagulation, tube morphogenesis, epidermis development, ectoderm development, embryonic development, neuron development, axonogenesis, ossification; **Table 4**). However, T+G+M activated genes involved in complement activation (i.e. Cfd, C4bp, Cfh, and Cd55) and regulation of ossification processes (i.e. Enpp1, Mgp, Ptger4, and Spp1). Some of these genes are closely related with cardiac functions and will be discussed later.

Compared with T+G+M, T+M failed to activate some cardiac-related processes (e.g. muscle development, regulation of heart contraction, structural constituent of muscle, glucose metabolism) and activated more genes involved in non-cardiac processes (e.g. morphogenesis of an epithelium, humoral immune response, axon guidance, blood vessel development and neuron development; **Table 2**). Additionally, T+M failed to inhibit genes involved in non-cardiac processes that were inhibited by T+G+M (e.g. embryonic limb morphogenesis, embryonic appendage morphogenesis, blood coagulation, ossification, humoral immune response; **Table 4**).

### Genes that underlay specific cardiac gene activation induced by Tbx5+Gata4+Myocd

As the genes activated or inhibited by T+G+M are not a simple plus of the genes activated or inhibited by Tbx5, Gata4, or Myocd alone, we tried to find out the genes that were specifically activated or inhibited by T+G+M by excluding genes activated or inhibited by Tbx5, Gata4, Myocd, T+G, G+M, and T+M from the activation or inhibition gene list of T+G+M. We found that T+G+M specifically activated 213 genes and inhibited 119 genes (**Supplemental Table S4** and **Supplemental Table S5**). In the specifically activated gene list by T+G+M, cardiac-related structural genes were Ldb3, Hsbp7, Myl4, Tnni3, Tnnc1, Mybpc3, Des, Myom2, Myh7 and Dsp. And cardiac-related transcription factors were Mlf1, Rbl1 and Atf6. In the specifically inhibited gene list by T+G+M, Cited2, Foxp1, Elk3 and Fgfr1 are known to be related with multiple developmental processes which will be discussed later. In the specifically activated and inhibited gene lists (**Supplemental Table S4** and **Supplemental Table S5**), there are several genes with unknown functions.

## Discussion

In our study, we found that Nkx2-5, Tbx5, Gata4, or Myocd alone did not induce the *de novo* expression of cardiac marker proteins in mouse 10T1/2 non-myoblastic cells. T+M and T+G+M were the only two combinations that were able to induce cardiac marker protein expression. The observation that Nkx2-5 was unhelpful in inducing Myh6 and Tnnt2 cardiac marker protein expression (**Table 1**) implies that Nkx2-5 does not contribute to or might inhibit cardiac gene activation, which is similar to previously reported observations [6], [7]. Microarray analysis revealed that Tbx5, Gata4, and Myocd play different roles in activating and inhibiting cardiac and non-cardiac gene expression. The combination of T+G+M activated the most genes involved in cardiac-related processes and the least genes involved in non-cardiac related processes. The Wnt receptor signaling pathway is known to be involved in every aspect of embryonic development, particularly in cardiac development and differentiation [32]. In T+G+M combination, the addition of Tbx5 (which singals activation of the Wnt receptor signaling pathway) may possibly: (1) balance with Myocd (which signals inhibition of the Wnt receptor signaling pathway); (2) remove the inhibitory effects of Gata4 on Myocd-induced cardiac-related process activation; and (3) activate additional cardiac-related processes including striated muscle contraction, regulation of heart contraction, structural constituents of muscle, and glucose metabolism (**Table 2**). These effects may be attributed to interactive modulations through physical interaction between Tbx5 and Myocd [18]. Myocd plays fundamental roles in muscle differentiation [4], [5], [13]. It is likely that Tbx5 helped to switch Myocd-induced muscle differentiation to cardiac side. This point was recently demonstrated by Wang and colleagues [18], who found that the direct interaction between Myocd and Tbx5 activated cardiac marker gene expression through Tbx5 binding sites [18]. The addition of Gata4 helped to enhance cardiac gene activation (i.e. regulation of heart contraction, structural constituents of muscle, and glucose metabolism) and inhibit non-cardiac gene expression (i.e. morphogenesis of an epithelium, humoral immune response, axon guidance, blood vessel development, and neuron development), which was activated by T+M (**Table 2**). The enhancement of cardiac gene activation by Gata4 may be attributed to physical interaction between Tbx5 and Gata4 [17]. In the present experimental setting, cardiac gene activation induced by T+G+M seemed unrelated with the activation of earlier heart developmental process, which was evidenced by the non-activation of cardiac mesoderm marker Mesp1 and second-heart field marker Isl1. This observation is similar to that reported by Ieda and his colleagues [7], which implied direct effects of Tbx5, Gata4, and Myocd on cardiac gene activation. Although Tbx5, Gata4, and Myocd are normally expressed in adult mouse heart (**Supplemental Figure S4**), forced expression of exogenous human Tbx5+Myocd or human Tbx5+Gata4+Myocd in 10T1/2 fibroblasts did not activate endogenous mouse Tbx5, Gata4, or Myocd expression in 10T1/2 fibroblasts (**Supplemental Table S3**). This is somehow similar to the previously reported observation showing that after the addition of exogenous Tbx5, Gata4, and Mef2c, endogenous Tbx5 and Mef2c expression was not induced [7]. Regarding exogenous human Tbx5, Gata4, and Myocd expression levels in Tbx5^+^Myocd^+^ or Tbx5^+^Gata4^+^Myocd^+^ 10T1/2 fibroblasts, it is difficult to give an exact answer. Unlike the uniform gene expression profile and biologic characteristics of individual ventricular cardiomyocytes, the expression levels of exogenous Tbx5, Gata4, and Myocd in Tbx5+Myocd or Tbx5+Gata4+Myocd transfected 10T1/2 fibroblasts may vary from cell to cell because of different copy numbers of the transgene integrated in the genome. Rare but appropriate expression levels and mutual expression ratios of exogenous Tbx5, Gata4, and Myocd may possibly account for the observation that only a small fraction (5.87–7.40%) of Tbx5^+^Gata4^+^Myocd^+^ 10T1/2 fibroblasts expressed cardiac marker proteins (**Table 1**). Therefore, comparing exogenous Tbx5, Gata4, and Myocd expression levels in Tbx5^+^Gata4^+^Myocd^+^/Myh6^+^ 10T1/2 fibroblasts with endogenous Tbx5, Gata4 and Myocd expression levels in mouse cardiomyocytes may provide valuable information regarding cardiac marker protein activation. To do this, we would need to combination the use of four fluorescence and drug-selection strategies (e.g. RFP for Tbx5, EGFP for Gata4, EBFP for Myocd, and a neomycin selection for the Myh6 reporter) in the future study. According to recent literature, it is controversial and confusing whether combination use of cardiac transcription factors Tbx5, Gata4, and Mef2c can reprogram mouse tail tip and cardiac fibroblasts into beating cardiomyocytes [7], [10]. Inconsistency also exists between Tbx5+Gata4+Mef2c and the recently reported Tbx5+Gata4+Mef2c+Hand2 combinations in their ability to generate beating cardiomyocytes [7], [9]. These inconsistencies may be related in part to the potential difference in tail tip and cardiac fibroblast isolation [10]. Therefore, it is important to use a reliable and reproducible mammalian non-myoblastic cell model to elucidate the individual roles of each cardiac transcription factors in activating cardiac gene expression. The information acquired can be very important for cardiac reprogramming and be more likely to be translated to other types of non-myoblastic cells. For this reason, we chose the mouse 10T1/2 fibroblast cell line to evaluate cardiac gene activation ability of several important cardiac transcription factors Nkx2-5, Tbx5, Gata4 and Myocd. Recently, our findings regarding the combination of T+G+M in potentiating cardiac gene and cardiac sarcomeric protein (Myh6) expression have been confirmed independently by Dr. Michael Schneider's group from Imperial College London (United Kingdom) in both mouse tail tip fibroblasts and Sca-1^+^ cardiac progenitor cells (unpublished observations; abstract submitted to 2012 American Heart Association Scientific Sessions).

Although T+G+M and T+M up-regulated Smarcd3 and Mef2c expression by 1.69- and 2.73-fold, respectively, no beating cardiomyocytes were observed in our study during a 4-week observation period. In consideration of the recent report that Tbx5+Gata4+Mef2c was inefficient to reprogram fibroblasts into cardiomyocytes [10], it is likely that Tbx5+Gata4+Mef2c may at least not function in all mammalian non-myoblastic cells and that its efficacy may be dependent on specific genetic, epigenetic, or transcriptome status of the candidate cells. Cardiac transcription factors Nkx2-5, Tbx5, Gata4, and Myocd regulate target genes in a cooperative way [17]–[20], [24]–[25], [28]. Several studies have indicated that a transcription factor alone is often less likely to potentiate target gene expression [2], [3], [18]. Although multiple binding sites (NKE, TBE, GATA box, and SRE) for Nkx2-5, Tbx5, Gata4, and SRF have been shown to exist in the promoter regions of target genes such as Myh6 and Nppa, it is difficult to tell exactly which genes are specific targets of a transcription factor. According to our array results (**Supplemental Table S2**), both Myh6 and Nppa are not activated by Tbx5, Gata4, or Myocd alone. However, Myh6 and Nppa are activated by the combination of Tbx5 and Myocd, which is consistent with the observations of Wang and colleagues [18]. Myocd is transcriptional cofactor for Srf [4] and is capable of activating both cardiac- and smooth muscle-specific gene expression, depending on the transcription factors (Srf, Tbx5) that it associates with [4], [18]. Because Srf is ubiquitously expressed in many cell types, the commonly observed ability of Myocd to activate smooth muscle gene is actually Srf-based. Smooth muscle myosin heavy chain (SM-MHC, also known as Myh11) can be regarded as a specific downstream target of Myocd in that its activation by Myocd is not affected by the addition of Tbx5 [18]. Our microarray results show that Myocd alone up-regulates Myh11 expression by 15.7 folds. In summary, the over-expression of Tbx5, Gata4, and Myocd not only induces the corresponding promoter activities (**Figure 1**) but also up-regulates target gene expression.

Although T+G+M activated genes that are related with complement activation (e.g. Cd55) and regulation of ossification (e.g. Spp1 and Ptger4) processes, these genes are also closely related with cardiac functions. Cd55 is a normally expressed cardiomyocyte receptor for Coxsackie B viruses [33]. Spp1, also known as osteopontin, is a multifunctional cytokine and adhesive protein that is important for cell-matrix and cell-cell interactions. The loss of Spp1 inhibits cardiomyocyte compartment function, which results in reduced cardiac performance [34]. Ptger4 mutation leads to patent ductus arteriosus [35]. If these genes are considered as cardiac-related rather than non-cardiac related, the non-cardiac processes of complement activation and regulation of ossification do not meet the activation criterion (nested gene change number ≥4). An interesting question is whether the gene expression profile in T+M or T+G+M transfected 10T1/2 fibroblasts is similar to that in cardiomyocytes. Considering that Tbx5^+^Gata4^+^Myocd^+^ 10T1/2 fibroblasts (including Tbx5^+^Gata4^+^Myocd^+^/Myh6^+^ subpopulation) are non-beating cells, we assume that the whole-genome gene expression profile may be different between Tbx5^+^Myocd^+^ or Tbx5^+^Gata4^+^Myocd^+^ 10T1/2 fibroblasts and mouse adult cardiomyocytes, although cardiac structural genes are activated in Tbx5^+^Myocd^+^ or Tbx5^+^Gata4^+^Myocd^+^ 10T1/2 fibroblasts (**Figure 2** and **Figure 3**). We conducted mouse heart microarray analysis and compared the results with those of Tbx5^+^Gata4^+^Myocd^+^ and Tbx5^+^Myocd^+^ 10T1/2 fibroblasts. Approximately 70% of whole-genome genes and 50% of cardiac cluster genes in T+G+M transfected 10T1/2 fibroblasts displayed trends similar to those in adult mouse heart (**Supplemental Figure S5**). Differentially expressed (activated or inhibited) genes (**a**, **b**, **c**, **d**, and **e** regions in **Supplemental Figure S5**) are related to cardiovascular system development. Region **(a)** genes are also related to striated muscle proliferation. The different expression patterns of these genes in T+M or T+G+M transfected 10T1/2 fibroblasts indicate the extensive perturbation of the cardiogenic process by the combination of Tbx5, Gata4, and Myocd.

In the list of genes specifically activated by T+G+M (**Supplemental Table S4**), Ldb3 (LIM domain binding 3, also known as Cypher) and Dsp (desmoplakin) are important for cardiomyocyte sarcomere development. Defects in either of these genes are associated with severe cardiomyopathies [36], [37]. The transcription factors Rbl1 (retinoblastoma-like 1, also known as p107), Atf6 (activating transcription factor 6), and Mlf1 (myeloid leukemia factor 1) are cardiac-related; they are expressed in the heart [38]–[40] and are involved in myogenic differentiation [39], Srf-mediated transcriptional regulation [40], and some unknown cardiac-related processes [38]. In the list of genes that are specifically inhibited by T+G+M (**Supplemental Table S5**), Cited2 (Cbp/p300-interacting transactivator), Foxp1 (Forkhead box P1), and Elk3 (member of the ETS oncogene family) are transcription factors that contribute to multiple developmental processes such as blood vessel development (Cited2) [41], nerve system development (Cited2) [42], embryonic placenta development (Cited2) [43], angiogenesis (Elk3) [44], lung development (Foxp1) [45], skeletal muscle development (Foxp1) [45], smooth muscle development (Foxp1) [45] and B-cell development (Foxp1) [46]. Additionally, Fgfr1 (fibroblast growth factor receptor 1), which was specifically inhibited by T+G+M, is related to nerve system development [47], angiogenesis [48], chondrocyte differentiation [49], lung development [50], embryonic limb morphogenesis [51], and inner ear morphogenesis [52]. These specifically activated and inhibited genes were most likely responsible for the specific cardiac gene activation spectrum induced by T+G+M. These findings further our understanding of transcription factor-based cardiac gene activation.

## Supporting Information

Figure S1 **Tracking multigene transfection at the single cell level.** CV-1 cells were infected with Nkx2-5, Tbx5, Gata4, Myocd lentivirus alone or some combinations. Cells were directly observed under confocal microscope 72 hours after doxycycline induction. Scale bar = 20 µm. N, Nkx2-5; T, Tbx5; G, Gata4; M, Myocd. DAPI was used to visualize nucleus.
(JPG)

Figure S2 **Molecular weights of the induced Myh6 and Tnnt2 proteins in 10T1/2 fibroblasts are same as those in mouse heart.** Tbx5+Myocd and Tbx5+Gata4+Myocd transfected 10T1/2 fibroblasts are induced by doxycycline (1 µg/ml) for 48 hours and are subjected to RFP^+^ and EGFP^+^ double selection by fluorescence-activated cell sorting. Sorted cells are induced by doxycycline (1 µg/ml) for additional 12 days followed by western blot analysis. The purity of Tbx5^+^Gata4^+^Myocd^+^ cells is 46.2% as confirmed under fluorescent microscope. LacZ transfected 10T1/2 fibroblasts and mouse heart are used as negative and positive controls, respectively. TM, Tbx5+Myocd; TGM, Tbx5+Gata4+Myocd. 4–20% SDS-PAGE.
(JPG)

Figure S3 **Quantitative reverse transcription polymerase chain reaction (qRT-PCR) analysis of cardiac marker gene expression in Tbx5, Gata4 and Myocd transfected 10T1/2 fibroblasts.** Cardiac marker gene Myh6, Ryr2, Nppa and Tnnt2 expression were examined in microarray leftover samples. Each experiment was performed in triplicate. T, Tbx5; G, Gata4; M, Myocd. The copy number for each transcript is expressed relative to that of glyceraldehyde-3-phosphate dehydrogenase (GAPDH), used as a constitutive control.
(JPG)

Figure S4 Reverse transcription polymerase chain reaction (RT-PCR) analysis of cardiac transcription factor expression in adult mouse heart.
(JPG)

Figure S5 **Difference exists in whole genome and cardiac cluster gene expression profiles between Tbx5+Gata4+Myocd transfected 10T1/2 fibroblasts and adult mouse myocardium.** (**A**) Whole genome gene expression profile. (**B**) Cardiac cluster gene expression profile. Cardiac genes are selected by their annotation containing “heart” or “cardiac”. Genes in differential expression regions (**a**), (**b**), (**c**), (**d**) and (**e**) are listed. Mouse hearts include biological duplicates (C57BL/6, male, 3 months of age). TM, T+M; TGM, T+G+M.
(JPG)

Table S1 **Activated and inhibited gene lists by Tbx5, Gata4, Myocd alone or different combinations.** Activated or inhibited genes were defined as genes with an absolute fold change ≥2.0 relative to the LacZ group and genes with a signal intensity equals to 0 in the LacZ group but not in the transcription factor-treated groups or vice versa.
(XLS)

Table S2 **Array signal values of several well-known cardiac marker genes in 10T1/2 fibroblasts.**
(DOC)

Table S3 **Array signal values of endogenous Nkx2-5, Tbx5, Gata4 and Myocd in 10T1/2 fibroblasts.**
(DOC)

Table S4 **Genes specifically activated by Tbx5+Gata4+Myocd in mouse 10T1/2 fibroblasts.** Tbx5+Gata4+Myocd specifically activated gene list was generated by excluding genes activated by Tbx5, Gata4, Myocd, T+G, G+M, and T+M from the activated gene list of T+G+M. Gene lists were compared by using the GeneVenn web application. * Gene accession number was used if probe set does not have a gene symbol.
(DOC)

Table S5 **Genes specifically inhibited by Tbx5+Gata4+Myocd in mouse 10T1/2 fibroblasts.** Tbx5+Gata4+Myocd specifically inhibited gene list was generated by excluding genes inhibited by Tbx5, Gata4, Myocd, T+G, G+M, and T+M from the inhibited gene list of T+G+M. Gene lists were compared by using the GeneVenn web application. * Gene accession number was used if probe set does not have a gene symbol.
(DOC)
