# Supplementary figures and images for: Cardiac Gene Activation Analysis in Mammalian Non-Myoblasic Cells by Nkx2-5, Tbx5, Gata4 and Myocd
Source: PLoS One. 2012 Oct 29;7(10):e48028. doi: 10.1371/journal.pone.0048028 (PMC3483304; doi:10.1371/journal.pone.0048028)

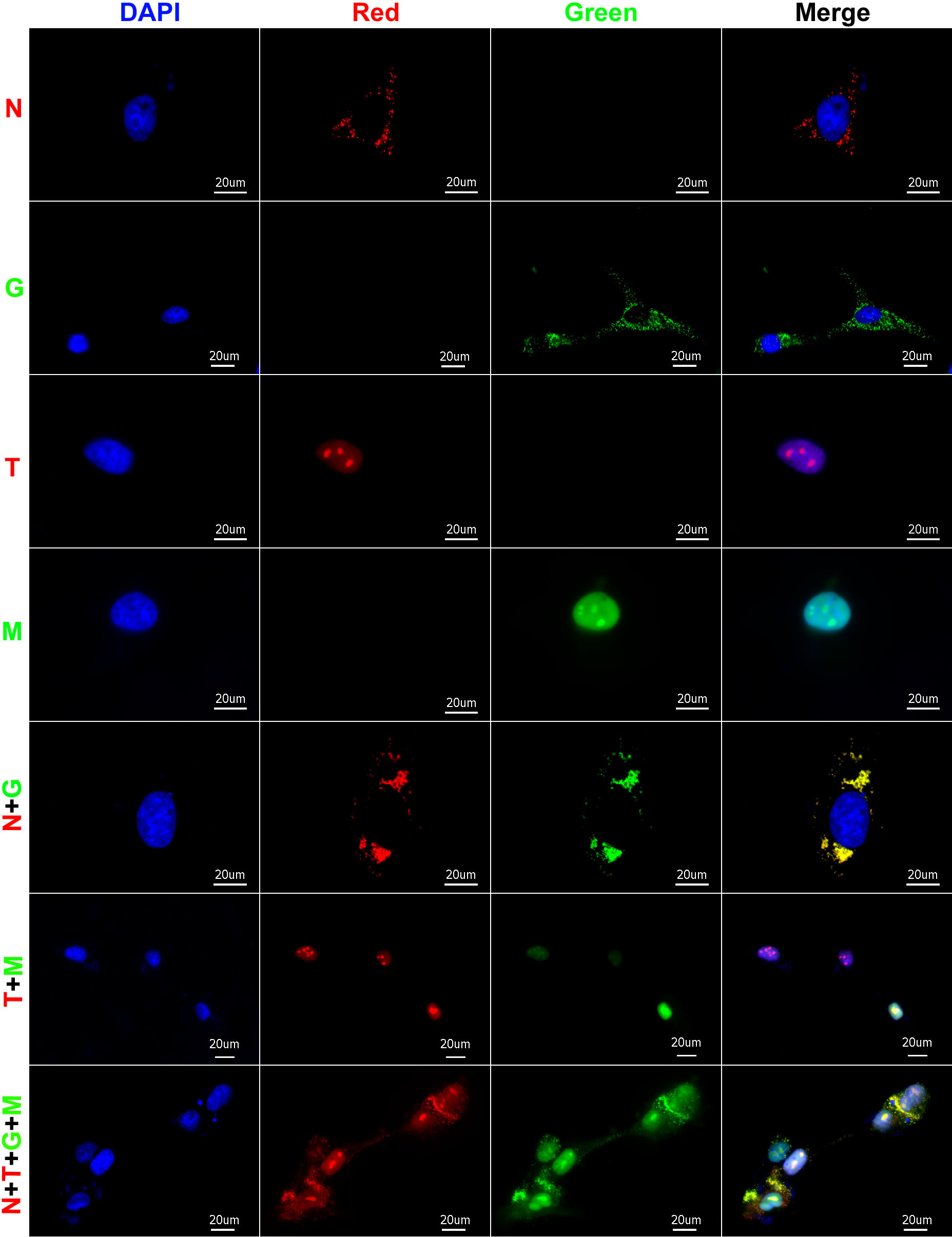

Supplement: Figure S1 — Tracking multigene transfection at the single cell level. CV-1 cells were infected with Nkx2-5, Tbx5, Gata4, Myocd lentivirus alone or some combinations. Cells were directly observed under confocal microscope 72 hours after doxycycline induction. Scale bar = 20 µm. N, Nkx2-5; T, Tbx5; G, Gata4; M, Myocd. DAPI was used to visualize nucleus. (JPG) [file pone.0048028.s001.jpg]

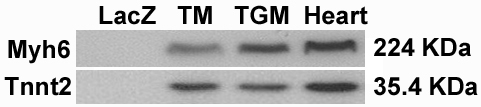

Supplement: Figure S2 — Molecular weights of the induced Myh6 and Tnnt2 proteins in 10T1/2 fibroblasts are same as those in mouse heart. Tbx5+Myocd and Tbx5+Gata4+Myocd transfected 10T1/2 fibroblasts are induced by doxycycline (1 µg/ml) for 48 hours and are subjected to RFP+ and EGFP+ double selection by fluorescence-activated cell sorting. Sorted cells are induced by doxycycline (1 µg/ml) for additional 12 days followed by western blot analysis. The purity of Tbx5+Gata4+Myocd+ cells is 46.2% as confirmed under fluorescent microscope. LacZ transfected 10T1/2 fibroblasts and mouse heart are used as negative and positive controls, respectively. TM, Tbx5+Myocd; TGM, Tbx5+Gata4+Myocd. 4–20% SDS-PAGE. (JPG) [file pone.0048028.s002.jpg]

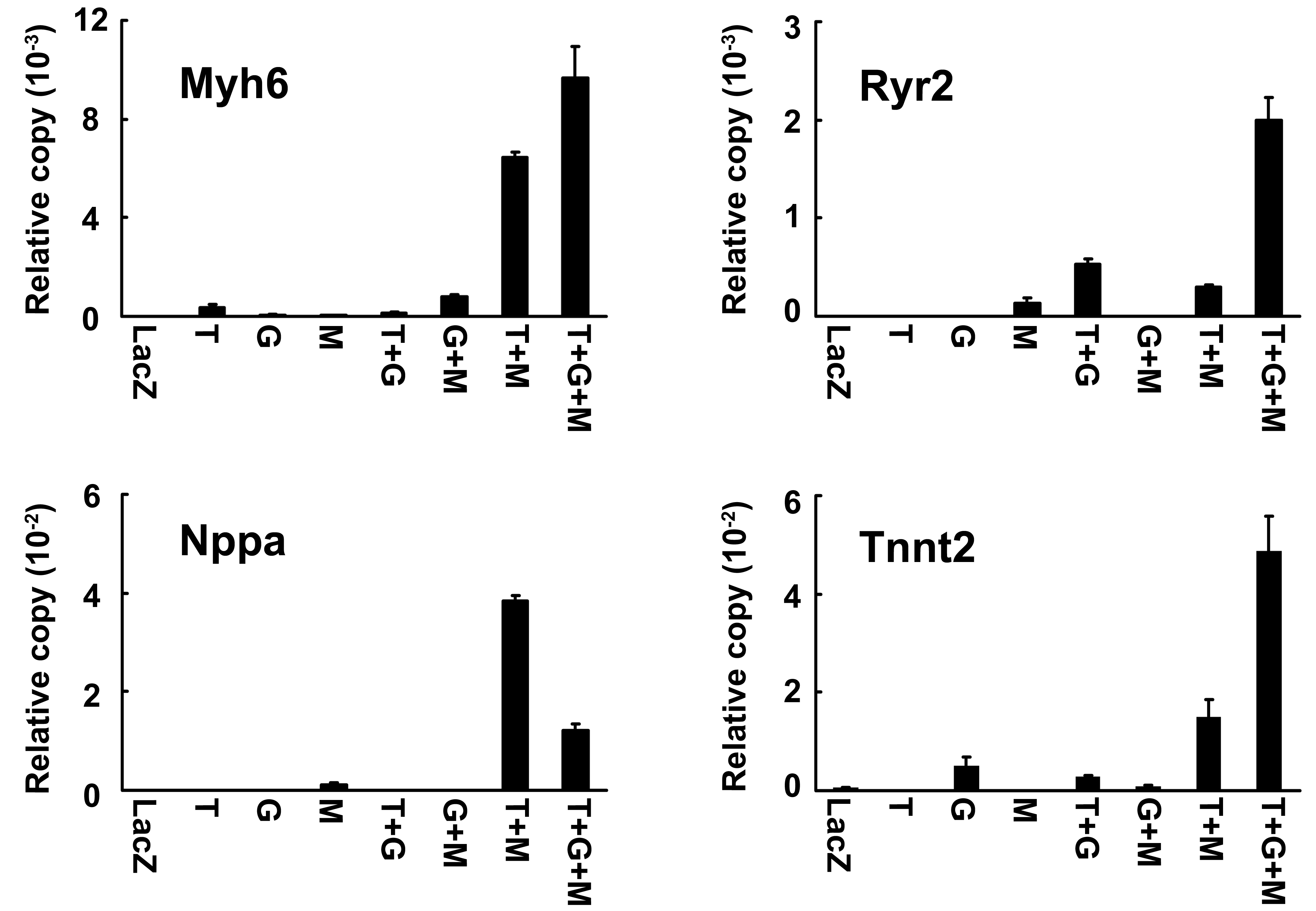

Supplement: Figure S3 — Quantitative reverse transcription polymerase chain reaction (qRT-PCR) analysis of cardiac marker gene expression in Tbx5, Gata4 and Myocd transfected 10T1/2 fibroblasts. Cardiac marker gene Myh6, Ryr2, Nppa and Tnnt2 expression were examined in microarray leftover samples. Each experiment was performed in triplicate. T, Tbx5; G, Gata4; M, Myocd. The copy number for each transcript is expressed relative to that of glyceraldehyde-3-phosphate dehydrogenase (GAPDH), used as a constitutive control. (JPG) [file pone.0048028.s003.jpg]

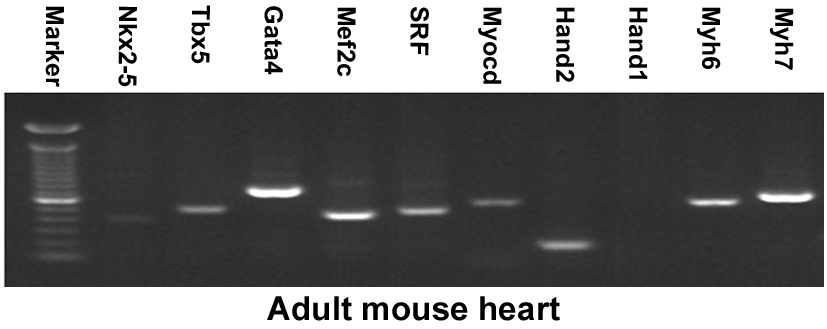

Supplement: Figure S4 — Reverse transcription polymerase chain reaction (RT-PCR) analysis of cardiac transcription factor expression in adult mouse heart. (JPG) [file pone.0048028.s004.jpg]

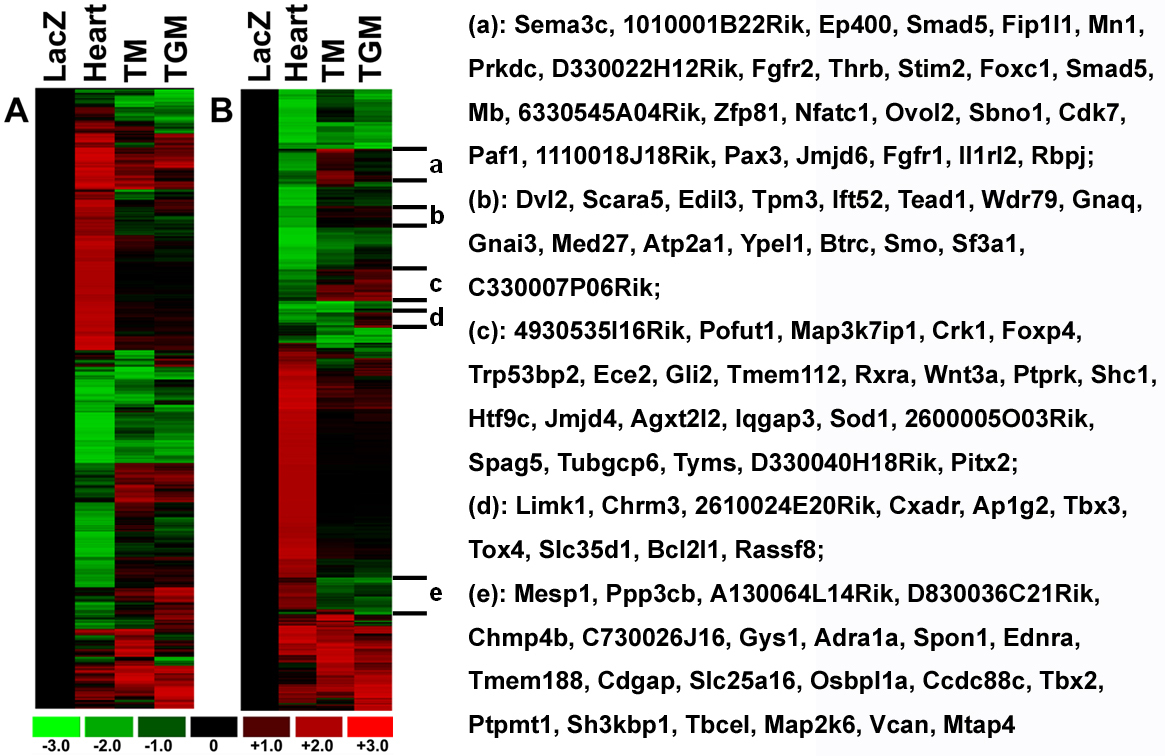

Supplement: Figure S5 — Difference exists in whole genome and cardiac cluster gene expression profiles between Tbx5+Gata4+Myocd transfected 10T1/2 fibroblasts and adult mouse myocardium. (A) Whole genome gene expression profile. (B) Cardiac cluster gene expression profile. Cardiac genes are selected by their annotation containing “heart” or “cardiac”. Genes in differential expression regions (a), (b), (c), (d) and (e) are listed. Mouse hearts include biological duplicates (C57BL/6, male, 3 months of age). TM, T+M; TGM, T+G+M. (JPG) [file pone.0048028.s005.jpg]
