# Supplementary material for: Cardiac Gene Activation Analysis in Mammalian Non-Myoblasic Cells by Nkx2-5, Tbx5, Gata4 and Myocd
Source: PLoS One. 2012 Oct 29;7(10):e48028. doi: 10.1371/journal.pone.0048028 (PMC3483304; doi:10.1371/journal.pone.0048028)
Supplement: Table S2 — Array signal values of several well-known cardiac marker genes in 10T1/2 fibroblasts. (DOC) [file pone.0048028.s007.doc]

**Supplemental Table S2. Array signal values of several well-known cardiac marker genes in 10T1/2 fibroblasts.**

| Cardiac markers | LacZ | Tbx5 | Gata4 | Myocd | TG | GM | TM | TGM |
| --- | --- | --- | --- | --- | --- | --- | --- | --- |
| Myh6 | 2.33 | 0.33 | 1.0 | 1.67 | 0.67 | 1.0 | 7.33 | 5.67 |
| Tnnt2 | 0 | 0 | 22.67 | 0.67 | 20.67 | 25.33 | 4.33 | 92.33 |
| Ryr2 | 0.67 | 0.67 | 0.67 | 1.33 | 1.67 | 0.67 | 1.67 | 2.67 |
| Nppa | 0 | 0 | 0 | 0 | 0 | 0 | 114.33 | 4.33 |
