# Supplementary material for: Cardiac Gene Activation Analysis in Mammalian Non-Myoblasic Cells by Nkx2-5, Tbx5, Gata4 and Myocd
Source: PLoS One. 2012 Oct 29;7(10):e48028. doi: 10.1371/journal.pone.0048028 (PMC3483304; doi:10.1371/journal.pone.0048028)
Supplement: Table S3 — Array signal values of endogenous Nkx2-5, Tbx5, Gata4 and Myocd in 10T1/2 fibroblasts. (DOC) [file pone.0048028.s008.doc]

**Supplemental Table S3.** Array signal values of endogenous Nkx2-5, Tbx5, Gata4 and Myocd in 10T1/2 fibroblasts.

| Transcription Factors | LacZ | Tbx5 | Gata4 | Myocd | TG | GM | TM | TGM |
| --- | --- | --- | --- | --- | --- | --- | --- | --- |
| Nkx2-5 | 70.33 | 89.67 | 70 | 103.33 | 89 | 75.33 | 86.67 | 90 |
| Tbx5 | 53.84 | 54.67 | 59.34 | 48.67 | 36.5 | 29.84 | 46.33 | 36.17 |
| Gata4 | 118.67 | 154.34 | 155.33 | 99.17 | 115.5 | 125.17 | 121.17 | 175.67 |
| Myocd | 42 | 56.17 | 35.67 | 61 | 52.5 | 53.5 | 45 | 37.67 |
