# Supplementary material for: Cardiac Gene Activation Analysis in Mammalian Non-Myoblasic Cells by Nkx2-5, Tbx5, Gata4 and Myocd
Source: PLoS One. 2012 Oct 29;7(10):e48028. doi: 10.1371/journal.pone.0048028 (PMC3483304; doi:10.1371/journal.pone.0048028)
Supplement: Table S4 — Genes specifically activated by Tbx5+Gata4+Myocd in mouse 10T1/2 fibroblasts. Tbx5+Gata4+Myocd specifically activated gene list was generated by excluding genes activated by Tbx5, Gata4, Myocd, T+G, G+M, and T+M from the activated gene list of T+G+M. Gene lists were compared by using the GeneVenn web application. * Gene accession number was used if probe set does not have a gene symbol. (DOC) [file pone.0048028.s009.doc]

**Supplemental Table S4. Genes specifically activated by Tbx5+Gata4+Myocd in mouse 10T1/2 fibroblasts**

| **Gene Symbol** | **Gene Title** |
| --- | --- |
| 1110059G02Rik | RIKEN cDNA 1110059G02 gene |
| 1500001M20Rik | RIKEN cDNA 1500001M20 gene |
| 1600002H07Rik | RIKEN cDNA 1600002H07 gene |
| 1700008F19Rik | RIKEN cDNA 1700008F19 gene |
| 1700049E17Rik1 | RIKEN cDNA 1700049E17 gene, gene 1 |
| 2410127L17Rik /// LOC677553 | RIKEN cDNA 2410127L17 gene /// similar to RIKEN cDNA 2410127L17 gene |
| 2610019F03Rik | RIKEN cDNA 2610019F03 gene |
| 2900026A02Rik | RIKEN cDNA 2900026A02 gene |
| 2900062L11Rik /// 6530401D17Rik | signal peptidase complex subunit 3 homolog pseudogene /// signal peptidase complex subunit 3 homolog pseudogene |
| 2900084O13Rik | RIKEN cDNA 2900084O13 gene |
| 3110001I22Rik | RIKEN cDNA 3110001I22 gene |
| 3632451O06Rik | RIKEN cDNA 3632451O06 gene |
| 3830403N18Rik /// Xlr | RIKEN cDNA 3830403N18 gene /// X-linked lymphocyte-regulated complex |
| 4930523O13Rik | RIKEN cDNA 4930523O13 gene |
| 4933402E13Rik | RIKEN cDNA 4933402E13 gene |
| Aaas | achalasia, adrenocortical insufficiency, alacrimia |
| Abca4 | ATP-binding cassette, sub-family A (ABC1), member 4 |
| Abhd6 | abhydrolase domain containing 6 |
| Afp | alpha fetoprotein |
| AI747699* | expressed sequence AI747699 |
| AK129341* | cDNA sequence AK129341 |
| Aldh1b1 | aldehyde dehydrogenase 1 family, member B1 |
| Ang4 | angiogenin, ribonuclease A family, member 4 |
| Ap1s3 | adaptor-related protein complex AP-1, sigma 3 |
| Aqp11 | aquaporin 11 |
| Areg | amphiregulin |
| Arhgap19 | Rho GTPase activating protein 19 |
| Arl4d | ADP-ribosylation factor-like 4D |
| Atad2 | ATPase family, AAA domain containing 2 |
| Atf6 | activating transcription factor 6 |
| Atg9b | ATG9 autophagy related 9 homolog B (S. cerevisiae) |
| Atp6v1c1 | ATPase, H+ transporting, lysosomal V1 subunit C1 |
| AW125035* | --- |
| AW986533* | --- |
| Baiap2l1 | BAI1-associated protein 2-like 1 |
| BB023692* | --- |
| BB148843* | --- |
| BB211194* | --- |
| BB353853* | --- |
| BB710847* | --- |
| BC030499* | cDNA sequence BC030499 |
| BC048355* | cDNA sequence BC048355 |
| BG060627* | --- |
| BG075777* | --- |
| BI076724* | --- |
| Bicd1 | bicaudal D homolog 1 (Drosophila) |
| BM115624* | --- |
| BM237858* | --- |
| Brip1 | BRCA1 interacting protein C-terminal helicase 1 |
| C2cd2 | C2 calcium-dependent domain containing 2 |
| C4bp | complement component 4 binding protein |
| C920025E04Rik | RIKEN cDNA C920025E04 gene |
| Ccdc141 | coiled-coil domain containing 141 |
| Ccne2 | cyclin E2 |
| Ccnf | cyclin F |
| Cd300a | CD300A antigen |
| Cdc6 | cell division cycle 6 homolog (S. cerevisiae) |
| Cdca5 | cell division cycle associated 5 |
| Cdt1 | chromatin licensing and DNA replication factor 1 |
| Cenpk | centromere protein K |
| Cenpq | centromere protein Q |
| Cep55 | centrosomal protein 55 |
| Ces2 /// Gm9756 | carboxylesterase 2 /// predicted gene 9756 |
| Chaf1b | chromatin assembly factor 1, subunit B (p60) |
| Chtf18 | CTF18, chromosome transmission fidelity factor 18 homolog (S. cerevisiae) |
| Clic5 | chloride intracellular channel 5 |
| Cma2 /// Mcpt9 | chymase 2, mast cell /// mast cell protease 9 |
| Cox6a2 | cytochrome c oxidase, subunit VI a, polypeptide 2 |
| Ctnnal1 | catenin (cadherin associated protein), alpha-like 1 |
| D030056L22Rik | RIKEN cDNA D030056L22 gene |
| D5Ertd505e | DNA segment, Chr 5, ERATO Doi 505, expressed |
| D930048N14Rik | RIKEN cDNA D930048N14 gene |
| Dctpp1 | dCTP pyrophosphatase 1 |
| Depdc7 | DEP domain containing 7 |
| Des | desmin |
| Dhfr | dihydrofolate reductase |
| Dnajc9 | DnaJ (Hsp40) homolog, subfamily C, member 9 |
| Dscc1 | defective in sister chromatid cohesion 1 homolog (S. cerevisiae) |
| Dsn1 | DSN1, MIND kinetochore complex component, homolog (S. cerevisiae) |
| Dsp | desmoplakin |
| Dtd1 /// LOC100048650 | D-tyrosyl-tRNA deacylase 1 homolog (S. cerevisiae) /// similar to D-tyrosyl-tRNA deacylase 1 |
| E2f8 | E2F transcription factor 8 |
| E330016A19Rik | RIKEN cDNA E330016A19 gene |
| Ear1 /// Ear12 /// Ear2 /// Ear3 | eosinophil-associated, ribonuclease A family, member 1 /// eosinophil-associated, ribonuclease A family, member 12 /// eosinophil-associated, ribonuclease A family, member 2 /// eosinophil-associated, ribonuclease A family, member 3 |
| Efna3 /// LOC100046031 | ephrin A3 /// similar to Ephrin A3 |
| Ehd4 | EH-domain containing 4 |
| Eif5a2 | eukaryotic translation initiation factor 5A2 |
| Enox1 | ecto-NOX disulfide-thiol exchanger 1 |
| Enpp4 | ectonucleotide pyrophosphatase/phosphodiesterase 4 |
| Esco2 | establishment of cohesion 1 homolog 2 (S. cerevisiae) |
| F11r | F11 receptor |
| Fam178b | family with sequence similarity 178, member B |
| Fam40b | family with sequence similarity 40, member B |
| Fbxo16 | F-box protein 16 |
| Fgfr3 | fibroblast growth factor receptor 3 |
| Fmc1 | formation of mitochondrial complexes 1 homolog (S. cerevisiae) |
| Fmo2 | flavin containing monooxygenase 2 |
| Foxa3 | forkhead box A3 |
| Fuca2 | fucosidase, alpha-L- 2, plasma |
| Fzd3 | frizzled homolog 3 (Drosophila) |
| Gas2l3 | growth arrest-specific 2 like 3 |
| Gm3916 | predicted gene 3916 |
| Gm5861 /// Gm6460 /// Speer1-ps1 | predicted gene 5861 /// predicted gene 6460 /// spermatogenesis associated glutamate (E)-rich protein 1, pseudogene 1 |
| Gpi1 | glucose phosphate isomerase 1 |
| Gpr123 | G protein-coupled receptor 123 |
| Gprc5c | G protein-coupled receptor, family C, group 5, member C |
| Gyk | glycerol kinase |
| Gzme | granzyme E |
| H2-D1 | histocompatibility 2, D region locus 1 |
| Hadhb | hydroxyacyl-Coenzyme A dehydrogenase/3-ketoacyl-Coenzyme A thiolase/enoyl-Coenzyme A hydratase (trifunctional protein), beta subunit |
| Hamp2 | hepcidin antimicrobial peptide 2 |
| Hbegf | heparin-binding EGF-like growth factor |
| Hdac6 | histone deacetylase 6 |
| Hells | helicase, lymphoid specific |
| Hirip3 | HIRA interacting protein 3 |
| Hoxc8 | homeo box C8 |
| Hspb7 | heat shock protein family, member 7 (cardiovascular) |
| Il17re | interleukin 17 receptor E |
| Insl6 | insulin-like 6 |
| Irak2 | interleukin-1 receptor-associated kinase 2 |
| Itih3 | inter-alpha trypsin inhibitor, heavy chain 3 |
| Kdm3a | lysine (K)-specific demethylase 3A |
| Klhl21 | kelch-like 21 (Drosophila) |
| Ldb2 | LIM domain binding 2 |
| Ldb3 | LIM domain binding 3 |
| Lgals3 | lectin, galactose binding, soluble 3 |
| Lims2 | LIM and senescent cell antigen like domains 2 |
| Lin9 | lin-9 homolog (C. elegans) |
| LOC100045677 /// Mcm3 | similar to DNA replication licensing factor MCM3 (DNA polymerase alpha holoenzyme-associated protein P1) (P1-MCM3) /// minichromosome maintenance deficient 3 (S. cerevisiae) |
| LOC552909 | hypothetical LOC552909 |
| Lonp1 | lon peptidase 1, mitochondrial |
| Mcm2 | minichromosome maintenance deficient 2 mitotin (S. cerevisiae) |
| Mcm4 | minichromosome maintenance deficient 4 homolog (S. cerevisiae) |
| Mcm6 | minichromosome maintenance deficient 6 (MIS5 homolog, S. pombe) (S. cerevisiae) |
| Mcm7 | minichromosome maintenance deficient 7 (S. cerevisiae) |
| Mcpt4 | mast cell protease 4 |
| Mlf1 | myeloid leukemia factor 1 |
| Mmp15 | matrix metallopeptidase 15 |
| Mpped2 | metallophosphoesterase domain containing 2 |
| Ms4a6b | membrane-spanning 4-domains, subfamily A, member 6B |
| Mybl1 | myeloblastosis oncogene-like 1 |
| Mybpc3 | myosin binding protein C, cardiac |
| Mycl1 | v-myc myelocytomatosis viral oncogene homolog 1, lung carcinoma derived (avian) |
| Myh7 | myosin, heavy polypeptide 7, cardiac muscle, beta |
| Myl4 | myosin, light polypeptide 4 |
| Myo5b | myosin VB |
| Myom2 | myomesin 2 |
| Nanos1 | nanos homolog 1 (Drosophila) |
| Ncf4 | neutrophil cytosolic factor 4 |
| Nkg7 | natural killer cell group 7 sequence |
| Nptx1 | neuronal pentraxin 1 |
| Nrarp | Notch-regulated ankyrin repeat protein |
| Nrm | nurim (nuclear envelope membrane protein) |
| Nup210 | nucleoporin 210 |
| Ormdl1 | ORM1-like 1 (S. cerevisiae) |
| Pard6b | par-6 (partitioning defective 6) homolog beta (C. elegans) |
| Pcdh20 | protocadherin 20 |
| Pde1c | phosphodiesterase 1C |
| Pde1c | phosphodiesterase 1C |
| Peli2 | pellino 2 |
| Plcb4 | phospholipase C, beta 4 |
| Plekhb2 | pleckstrin homology domain containing, family B (evectins) member 2 |
| Pola2 | polymerase (DNA directed), alpha 2 |
| Pon2 | paraoxonase 2 |
| Prl2c2 /// Prl2c3 /// Prl2c4 | prolactin family 2, subfamily c, member 2 /// prolactin family 2, subfamily c, member 3 /// prolactin family 2, subfamily c, member 4 |
| Prps2 | phosphoribosyl pyrophosphate synthetase 2 |
| Rad51ap1 | RAD51 associated protein 1 |
| Rad54b | RAD54 homolog B (S. cerevisiae) |
| Rad54l | RAD54 like (S. cerevisiae) |
| Rbl1 | retinoblastoma-like 1 (p107) |
| Rhox5 | reproductive homeobox 5 |
| Rin1 | Ras and Rab interactor 1 |
| Rinl | Ras and Rab interactor-like |
| Sac3d1 | SAC3 domain containing 1 |
| Sema3g | sema domain, immunoglobulin domain (Ig), short basic domain, secreted, (semaphorin) 3G |
| Serpina3m | serine (or cysteine) peptidase inhibitor, clade A, member 3M |
| Serpina3n | serine (or cysteine) peptidase inhibitor, clade A, member 3N |
| Serpina9 | serine (or cysteine) peptidase inhibitor, clade A (alpha-1 antiproteinase, antitrypsin), member 9 |
| Serpinb7 | serine (or cysteine) peptidase inhibitor, clade B, member 7 |
| Slbp | stem-loop binding protein |
| Slc25a33 | solute carrier family 25, member 33 |
| Slc6a18 | solute carrier family 6 (neurotransmitter transporter), member 18 |
| Slc6a19 | solute carrier family 6 (neurotransmitter transporter), member 19 |
| Stard8 | START domain containing 8 |
| Strbp | spermatid perinuclear RNA binding protein |
| Svip | small VCP/p97-interacting protein |
| Tac1 | tachykinin 1 |
| Taldo1 | transaldolase 1 |
| Tap1 | transporter 1, ATP-binding cassette, sub-family B (MDR/TAP) |
| Tcf19 | transcription factor 19 |
| Tcf23 | transcription factor 23 |
| Tchh | trichohyalin |
| Tec | tec protein tyrosine kinase |
| Tmsb15b1-Tmsb15b2 /// Tmsb15b2 | Tmsb15b1-Tmsb15b2 readthrough transcript /// thymosin beta 15b2 |
| Tmx4 | thioredoxin-related transmembrane protein 4 |
| Tnfaip8l1 | tumor necrosis factor, alpha-induced protein 8-like 1 |
| Tnfrsf12a | tumor necrosis factor receptor superfamily, member 12a |
| Tnfsf9 | tumor necrosis factor (ligand) superfamily, member 9 |
| Tnnc1 | troponin C, cardiac/slow skeletal |
| Tnni3 | troponin I, cardiac 3 |
| Trmt61b | tRNA methyltransferase 61 homolog B (S. cerevisiae) |
| Tspan8 | Tetraspanin 8 |
| Ube2t | ubiquitin-conjugating enzyme E2T (putative) |
| Ufsp1 | UFM1-specific peptidase 1 |
| Uhrf1 | ubiquitin-like, containing PHD and RING finger domains, 1 |
| Ung | uracil DNA glycosylase |
| Usp27x | ubiquitin specific peptidase 27, X chromosome |
| Vtn | vitronectin |
| Wdr47 | WD repeat domain 47 |
| Wfs1 | Wolfram syndrome 1 homolog (human) |
| Zfp133 | zinc finger protein 133 |
| Zfp667 | zinc finger protein 667 |
| Znrf2 | zinc and ring finger 2 |

* Gene accession number was used if probe set does not have a gene symbol.
