# Supplementary material for: Cardiac Gene Activation Analysis in Mammalian Non-Myoblasic Cells by Nkx2-5, Tbx5, Gata4 and Myocd
Source: PLoS One. 2012 Oct 29;7(10):e48028. doi: 10.1371/journal.pone.0048028 (PMC3483304; doi:10.1371/journal.pone.0048028)
Supplement: Table S5 — Genes specifically inhibited by Tbx5+Gata4+Myocd in mouse 10T1/2 fibroblasts. Tbx5+Gata4+Myocd specifically inhibited gene list was generated by excluding genes inhibited by Tbx5, Gata4, Myocd, T+G, G+M, and T+M from the inhibited gene list of T+G+M. Gene lists were compared by using the GeneVenn web application. * Gene accession number was used if probe set does not have a gene symbol. (DOC) [file pone.0048028.s010.doc]

**Supplemental Table S5. Genes specifically inhibited by Tbx5+Gata4+Myocd in mouse 10T1/2 fibroblasts**

| **Gene Symbol** | **Gene Title** |
| --- | --- |
| Sept6 | septin 6 |
| 1700052K11Rik | RIKEN cDNA 1700052K11 gene |
| 2310039F13Rik | RIKEN cDNA 2310039F13 gene |
| 3110027N22Rik | RIKEN cDNA 3110027N22 gene |
| 5930422O12Rik | RIKEN cDNA 5930422O12 gene |
| 9030425E11Rik | RIKEN cDNA 9030425E11 gene |
| 9430091N11Rik | RIKEN cDNA 9430091N11 gene |
| 9430098F02Rik | RIKEN cDNA 9430098F02 gene |
| A130010J15Rik | RIKEN cDNA A130010J15 gene |
| A430104N18Rik | RIKEN cDNA A430104N18 gene |
| Adamts1 | a disintegrin-like and metallopeptidase (reprolysin type) with thrombospondin type 1 motif, 1 |
| Adamts6 | a disintegrin-like and metallopeptidase (reprolysin type) with thrombospondin type 1 motif, 6 |
| Akap12 | A kinase (PRKA) anchor protein (gravin) 12 |
| Ankrd42 | ankyrin repeat domain 42 |
| Arl15 | ADP-ribosylation factor-like 15 |
| AU016662* | Transcribed locus |
| AU040128* | --- |
| AW550459* | Transcribed locus |
| AW824326* | Transcribed locus |
| B930095G15Rik | RIKEN cDNA B930095G15 gene |
| BB036443* | --- |
| BB253657* | Transcribed locus |
| BB409477* | 7 days embryo whole body cDNA, RIKEN full-length enriched library, clone:C430014D18 product:unclassifiable, full insert sequence |
| BB468566* | --- |
| BB472891* | --- |
| BB540658* | 0 day neonate eyeball cDNA, RIKEN full-length enriched library, clone:E130107G13 product:histocompatibility 2, T region locus 18, full insert sequence |
| BE953350* | Transcribed locus |
| BF472132* | Transcribed locus |
| BF715246* | --- |
| BG067231* | --- |
| BG069057* | --- |
| BG069620* | --- |
| BI499709* | Transcribed locus |
| BI901592* | Transcribed locus |
| BM232535* | Lung RCB-0558 LLC cDNA, RIKEN full-length enriched library, clone:G730023O13 product:unclassifiable, full insert sequence |
| Bmp15 | bone morphogenetic protein 15 |
| Bnc2 | basonuclin 2 |
| C1r | complement component 1, r subcomponent |
| C1s /// LOC100044326 | complement component 1, s subcomponent /// similar to Complement component 1, s subcomponent |
| C3 | complement component 3 |
| Cacna1c | calcium channel, voltage-dependent, L type, alpha 1C subunit |
| Cc2d1a | coiled-coil and C2 domain containing 1A |
| Ccdc102a | coiled-coil domain containing 102A |
| Ccdc80 | coiled-coil domain containing 80 |
| Cdc42bpb | CDC42 binding protein kinase beta |
| Cited2 | Cbp/p300-interacting transactivator, with Glu/Asp-rich carboxy-terminal domain, 2 |
| Clip3 | CAP-GLY domain containing linker protein 3 |
| Col11a1 | collagen, type XI, alpha 1 |
| Col27a1 | collagen, type XXVII, alpha 1 |
| Col5a1 | collagen, type V, alpha 1 |
| Col6a2 | collagen, type VI, alpha 2 |
| Cstad | CSA-conditional, T cell activation-dependent protein |
| Ctsf | cathepsin F |
| D0H4S114 | DNA segment, human D4S114 |
| D730035F11Rik | RIKEN cDNA D730035F11 gene |
| D930026N18Rik | RIKEN cDNA D930026N18 gene |
| Dhx58 | DEXH (Asp-Glu-X-His) box polypeptide 58 |
| Dpep1 | dipeptidase 1 (renal) |
| Ehbp1 | EH domain binding protein 1 |
| Elk3 | ELK3, member of ETS oncogene family |
| Emilin1 | elastin microfibril interfacer 1 |
| Fam189b | family with sequence similarity 189, member B |
| Fgfr1 | fibroblast growth factor receptor 1 |
| Foxn3 | forkhead box N3 |
| Foxp1 | Forkhead box P1 |
| Ftsj1 | FtsJ homolog 1 (E. coli) |
| Gm13305 /// Gm2002 /// Il11ra1 /// Il11ra2 | predicted gene 13305 /// predicted gene 2002 /// interleukin 11 receptor, alpha chain 1 /// interleukin 11 receptor, alpha chain 2 |
| Gpd2 | glycerol phosphate dehydrogenase 2, mitochondrial |
| Grb14 | growth factor receptor bound protein 14 |
| Hunk | hormonally upregulated Neu-associated kinase |
| Ier5l | immediate early response 5-like |
| Ifi44 | interferon-induced protein 44 |
| Ifnar2 | interferon (alpha and beta) receptor 2 |
| Irak3 | interleukin-1 receptor-associated kinase 3 |
| Kcns2 | K+ voltage-gated channel, subfamily S, 2 |
| Kcnt2 | potassium channel, subfamily T, member 2 |
| Klf15 | Kruppel-like factor 15 |
| Lbp | lipopolysaccharide binding protein |
| LOC552876 | hypothetical LOC552876 |
| Lox | lysyl oxidase |
| Lrp2bp | Lrp2 binding protein |
| Mmp2 | matrix metallopeptidase 2 |
| Mup1 /// Mup10 /// Mup2 | major urinary protein 1 /// major urinary protein 10 /// major urinary protein 2 |
| Mxra7 | matrix-remodelling associated 7 |
| Naf1 | nuclear assembly factor 1 homolog (S. cerevisiae) |
| Nek6 | NIMA (never in mitosis gene a)-related expressed kinase 6 |
| Nfkbia | nuclear factor of kappa light polypeptide gene enhancer in B-cells inhibitor, alpha |
| Nlrx1 | NLR family member X1 |
| NM_025367* | --- |
| Omd | osteomodulin |
| Pde1a | phosphodiesterase 1A, calmodulin-dependent |
| Pdgfrb | platelet derived growth factor receptor, beta polypeptide |
| Pdzd2 | PDZ domain containing 2 |
| Phex | phosphate regulating gene with homologies to endopeptidases on the X chromosome (hypophosphatemia, vitamin D resistant rickets) |
| Pias3 | protein inhibitor of activated STAT 3 |
| Plagl2 | pleiomorphic adenoma gene-like 2 |
| Pppde1 | PPPDE peptidase domain containing 1 |
| Prkcd | protein kinase C, delta |
| Ptgfr | prostaglandin F receptor |
| Purg | purine-rich element binding protein G |
| Pxdn | peroxidasin homolog (Drosophila) |
| Ramp2 | receptor (calcitonin) activity modifying protein 2 |
| Sat2 | spermidine/spermine N1-acetyl transferase 2 |
| Sema3a | sema domain, immunoglobulin domain (Ig), short basic domain, secreted, (semaphorin) 3A |
| Serf1 | small EDRK-rich factor 1 |
| Sh3pxd2b | SH3 and PX domains 2B |
| Snx18 | sorting nexin 18 |
| Src | Rous sarcoma oncogene |
| Stx2 | syntaxin 2 |
| Sulf1 | sulfatase 1 |
| Tbc1d2b | TBC1 domain family, member 2B |
| Tle2 | transducin-like enhancer of split 2, homolog of Drosophila E(spl) |
| Tle6 | transducin-like enhancer of split 6, homolog of Drosophila E(spl) |
| Tmco7 | transmembrane and coiled-coil domains 7 |
| Trpm1 | Transient receptor potential cation channel, subfamily M, member 1 |
| Tsc22d3 | TSC22 domain family, member 3 |
| Wipf1 | WAS/WASL interacting protein family, member 1 |
| Wrn | Werner syndrome homolog (human) |
| Zfp90 | zinc finger protein 90 |

* Gene accession number was used if probe set does not have a gene symbol.
